# NUT midline carcinoma in a young pregnant female: a case report

**DOI:** 10.1186/s12957-020-02065-6

**Published:** 2020-11-07

**Authors:** Sebastian Joel, Friederike Weschenfelder, Ekkehard Schleussner, Gunther O. Hofmann, Wolfram Weschenfelder

**Affiliations:** 1 Department of Trauma, Hand and Reconstructive Surgery, University Hospital Jena, Jena, Germany; 2 Department of Obstetrics, University Hospital Jena, Jena, Germany

**Keywords:** NUT midline carcinoma, Pregnancy, Soft tissue tumor, Orthopedic oncology, Interdisciplinarity

## Abstract

**Introduction:**

The NUT midline carcinoma is a rare tumor mostly reported in the midline of upper aerodigestive tract and mediastinum. Children as well as adolescents are affected without a gender distribution. A standard treatment is not established. So far, there exists no reported case of a pregnant female suffering from NUT midline carcinoma with musculoskeletal manifestation.

**Case presentation:**

A 34-year-old woman was referred to our outpatient clinic by the general practitioner during her 31st week of pregnancy suffering from shoulder pain and dyspnea. So far, dyspnea was interpreted as a typical pregnancy-related symptom. However, a chest X-ray showed a tumor mass in the right lung in close relation to the scapula. Further examinations found metastases in different areas of the body. No pregnancy-related complications were detected by obstetric examination. After an interdisciplinary perinatal case discussion, cesarean section was directly followed by an open biopsy of the right side scapula tumor lesion. A NUT midline carcinoma was diagnosed by immunohistochemistry. Due to disseminated tumor disease in multiple non-resectable locations, a palliative systemical chemotherapy was started by the oncological outpatient clinic.

**Conclusion:**

This report presents the case of the very rare NUT midline carcinoma under pregnancy which made interdisciplinary case discussions indispensable for therapy planning.

## Introduction

The NUT midline carcinoma is a rare tumor mostly reported in the midline of the skull, upper aerodigestive tract, and mediastinum [1–3]. Other parts of the body such as lung, abdominal organs, or bone can also be affected but cases are less common [4–6]. The first descriptions of intrathoracic high-grade carcinomas harboring the typical (15;19) translocation were published in 1991 by two different groups [7, 8]. The NUT midline carcinoma affects children as well as adolescents and adults with a balanced overall gender distribution. The outcome was reported with a 2-year overall survival of 19% and a median overall survival of 6.7 months [9]. Clinical symptoms are dependent on the special location of the tumor. Patients often complain about shortness of breath, bloody sputum, non-productive cough, chest pain, nausea, odynophagia, lymphadenopathy, lumbago, local swellings, history of fever, and sweating as well as weight loss [6, 10–15]. Blood tests revealed an unspecific leucocytosis [11]. So far a special blood tumor marker does not exist. After detecting a tumor mass by methods of radiological examination, usually a tumor biopsy is performed to enable a soft tissue analysis. NUT midline carcinomas are a poorly differentiated class of tumor but mostly show a typical (15;19) translocation in fluorescence in situ hybridization (FISH) [16]. There are several cases with cryptic translocation that do not show the typical NUT gene rearrangements by FISH [17]. As a further diagnostic test, there exists a characteristic monoclonal antibody to NUT genes which reaches a specificity of 100% [18]. For NUT midline carcinoma, there is no established specific therapy regimen until now. In different studies, patients received a multimodal therapy consisting of various combinations of surgery, chemotherapy, and radiotherapy [19]. Different outcomes and survival were reported because the chosen therapy regimen always depends on individual factors [19–21]. The use of BET (bromodomain and extra-terminal) protein inhibitors is a new targeting immunotherapy. It is applied alone or in combination with other therapeutic agents in patients with NUT midline carcinoma and an arrest of growth under this therapy has been reported [22].

## Case presentation

**Fig. 1:**
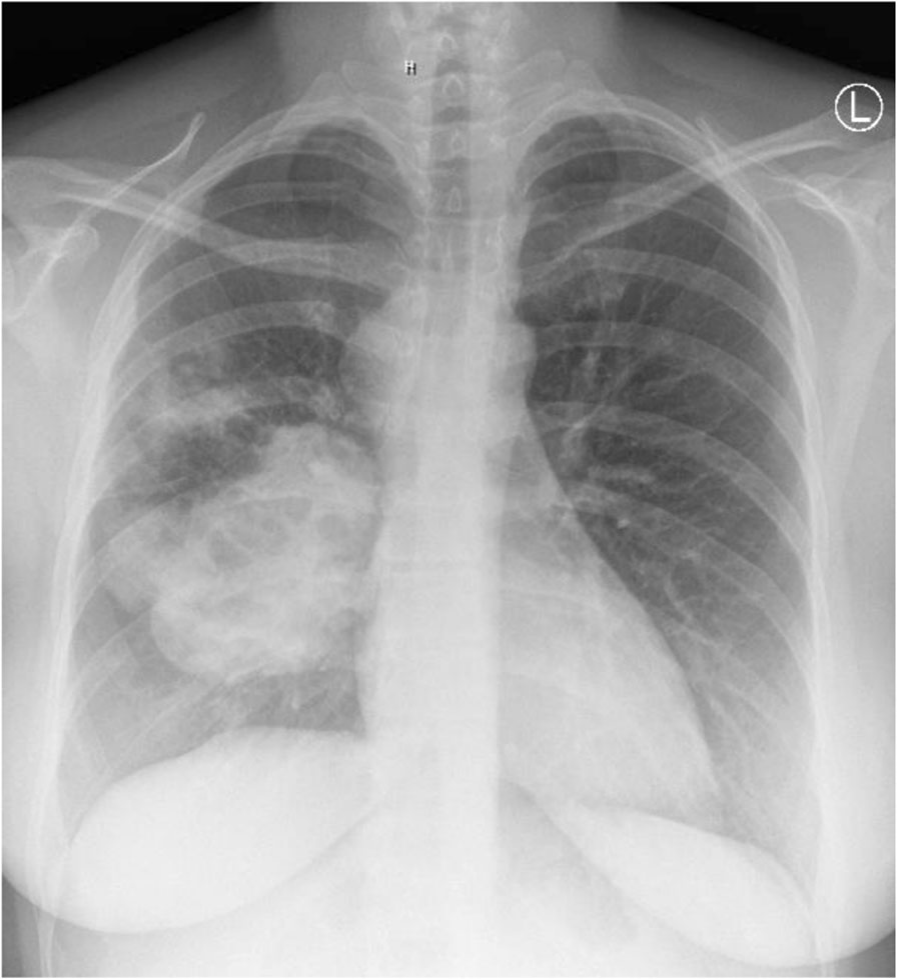
X-ray AP view of thorax showing tumor mass in the right lung

A 34-year-old woman was referred to our outpatient clinic during her 31st week of pregnancy suffering from shoulder pain of the right side after lifting her 11-month-old first born. During the first pregnancy, one and a half year ago she started noticing dyspnea which was still present at the time of her presentation in our clinic. At that time, this symptom was considered as pregnancy-related therefore no special examination was arranged. Several months later the patient became pregnant again. She was still reporting of dyspnea especially at physical activity and new symptoms, cough and hemoptysis, had appeared. Due to persistent symptoms, the general practitioner arranged a chest X-ray that showed a tumor mass in the right lung in close relation to the scapula (Fig. 1).

Clinical examination showed a patient in good general health. There was a local swelling at the medial border of the right scapula with dull pressure pain. The movement of the shoulder was painless at full range of motion and strength. The auscultation of the lung presented a vesicular respiratory sound which was slightly attenuated at the right side. The patient did not suffer from pain. Family anamnesis revealed a breast cancer of the patient’s mother and a bronchial carcinoma of the father. B symptoms were not reported.

The case was primarily presented to the department of obstetrics in 31 4/7 weeks of gestation. Ultrasound showed a large for gestational fetus (2205 g, 93rd percentile) with normal amniotic fluid and normal feto-placental perfusion of a 34-year-old gravida 2 para 1 (one spontaneous delivery 1 year ago) with no pregnancy-related complications. A diaplacental cancer cell transmission seemed unlikely for the type of expected tumor.

**Fig. 2:**
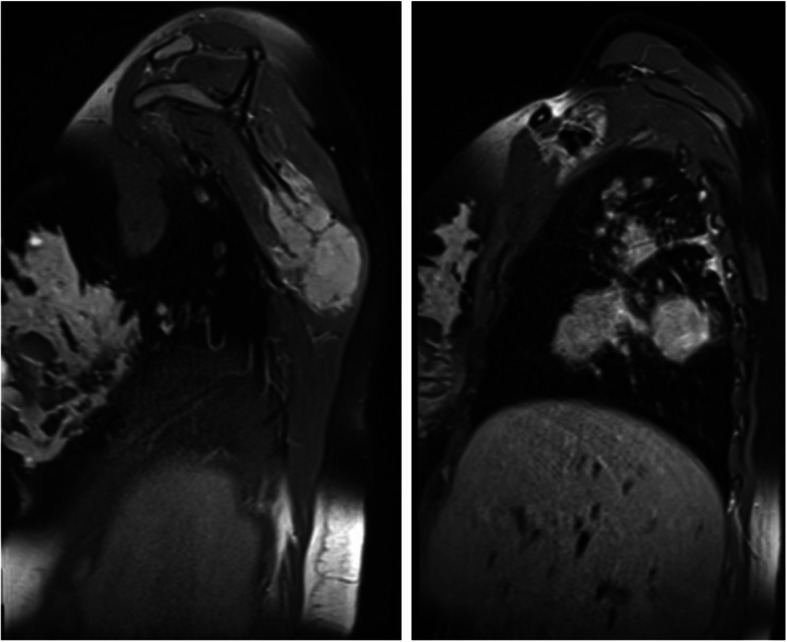
MRI sagittal view showing highly suspicious lesions in the right scapula and lung

MRI scans (magnetic resonance imaging, Fig. 2) of the cranium and the thorax were performed and revealed several highly suspicious lesions in the right lung, the mediastinum, the inferior angle of the right scapula with soft tissue component, and an osteolytic lesion of the 3rd thoracic vertebral body with affection of dura and spinal nerve.

An interdisciplinary perinatal case discussion with obstetricians, neonatologists, oncologists, and our orthopedic department was initiated near-term discussing the conflict of delaying maternal tumor therapy and causing an iatrogenic preterm birth and its possible neonatal complications. In 32 0/7 weeks of gestation, the pros and cons due to a delivery before 34 0/7 weeks of gestation were discussed and presented to the patient and her husband by the treating specialist, so that informed consent could be obtained. In summary, a one-stage procedure, combining the primary cesarean section and the open biopsy of the right side scapula tumor lesion was planned for 33 4/7 weeks of gestation. Before the planned delivery, antenatal corticosteroid treatment for accelerating fetal lung maturation was implemented to minimize the risk for respiratory neonatal complications. Thrombosis and pneumonia prophylaxis were recommended due to the high-risk situation.

A fine-needle biopsy of the pulmonary tumor mass was done by bronchoscopy. Subsequently, the cesarean section was directly followed by an open biopsy of the right side scapula tumor lesion. A NUT midline carcinoma was diagnosed by immunohistochemistry (Table 1) while FISH analysis did not show the typical NUT gene rearrangements due to cryptic translocation.

**Table 1:** Biopsy of pulmonary tumor and scapula tumor lesion

**Immunohistochemistry**	NUT+, p63+ CK20−, CDX2−, GATA3−, ER−, PR−, PD-L1−, CK5/6−, ALK−, ROS1−, PAX8−, S100−, Napsin−, Melan A−, P40− (mostly), HER2 score 2+

+ positive, − negative

**Fig. 3:**
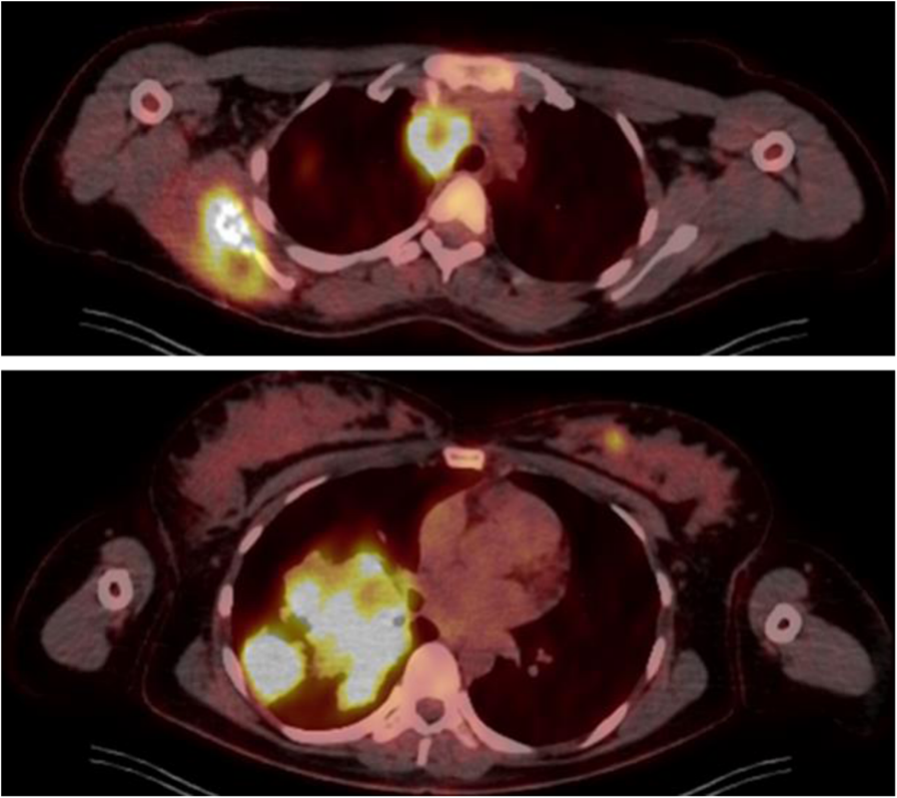
PET-CT axial view showing tumor lesions in the right scapula, mediastinum, and lung

**Fig. 4:**
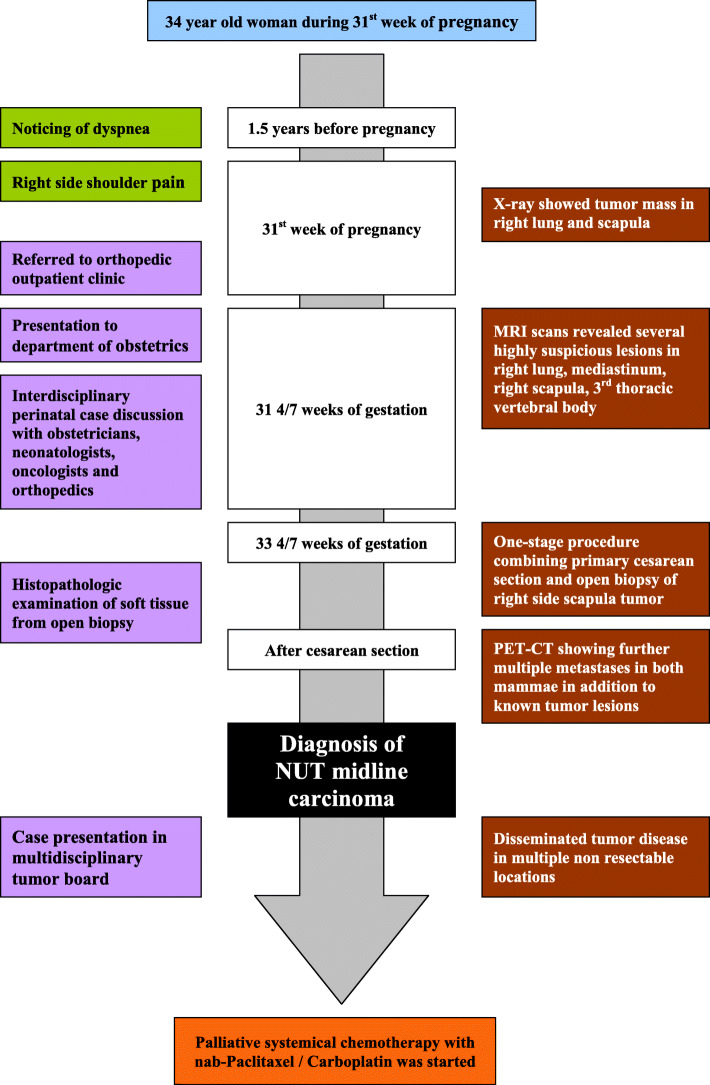
Timeline of the case

The examination of the placenta did not show any signs of malignancy. After the cesarean section, a PET-CT (positron emission tomography-computed tomography, Fig. 3) was performed showing further multiple metastases in both mammae in addition to the known tumor lesions. The case was presented in a multidisciplinary tumor board. Due to disseminated tumor disease in multiple non-resectable locations, a palliative systemical chemotherapy with nab-paclitaxel (100 mg/m^2^)/carboplatin (AUC 6) was started by the oncological outpatient clinic (Fig. 4). Priorly, Trenantone® 130 mg subcutaneously for ovary protection was administered. Furthermore, an osteoprotective bisphosphonate therapy with Zometa® 4 mg was begun. Regarding the bone metastases in the right scapula and in the 3rd thoracic vertebral body, no surgical intervention but radiotherapy was planned in the further course as part of the palliative therapy regimen. After five courses of systemical chemotherapy with nab-paclitaxel/carboplatin, the patient showed a decline of dyspnea. A PET-CT 3 months after the start of therapy demonstrated a partial remission of known metastases in the right lung and in both mammae. Systemical chemotherapy was continued by the oncological outpatient clinic. However, the scheduled PET-CT after 5 months showed a tumor progress with growth of known metastases and new lesions in the spine and pelvis. Again, the case was discussed in multidisciplinary tumor board and systemic treatment was changed to pembrolizumab while an indication for BET inhibitors was proved in cooperation with an external oncological clinic. Unfortunately, further progress of known metastases was demonstrated radiologically as well as new spinal and pelvic lesions. After that, the patient was lost for further follow-up and missed further appointments.

## Discussion and conclusions

Until now NUT midline carcinomas have been reported in children and adolescents as well as adults [9]. Young women around the age of 30 may be affected by this malignancy [15, 23]. However, this report presents the first published case of a NUT midline carcinoma in a non-smoking young female patient under pregnancy. In the recently published review of Dalmartello et al., the most common pregnancy-associated carcinomas were melanoma, breast cancer, cervical cancer, and thyroid cancer [24]. The placenta and tumor microenvironment seems to be extremely comparable, in particular, the immune responses for growth and vascularization as well as hormone sensitivity. Beaman et al. state the importance of leucocytes in activating and supporting tumor and placental cell growth through the production of growth factors and angiogenic factors [25]. For pregnancy-associated melanoma, even diaplacental transmission has been described and therefore careful examination of placentas of women with known or suspected metastatic melanoma is recommended [26].

The patient in this case had complained of dyspnea since her first pregnancy. In addition cough and hemoptysis had occurred as further symptoms during her second pregnancy. Dyspnea is a disorder often reported by pregnant women. Its persistence after labor is untypical and should have led to earlier diagnostic investigations. The symptoms reported by the patient, dyspnea, cough, and hemoptysis, are typical for severe illnesses of the lung and hence for a pulmonary manifestation of a NUT midline carcinoma. In addition, the patient had noted an unclear swelling of the soft tissues below the scapula over several weeks - a condition that is a typical clinical presentation of soft tissue tumors and has to be further verified by sonography and MRI.

In published cases of NUT midline carcinomas, the primary tumor localization was mostly common in the upper aerodigestive tract and mediastinum [1, 2] but the lung was also reported [15]. However, the primary localization in the presented case remains unclear. According to other publications, complete surgical resection is associated with improved overall survival [19]. This could not be performed in the presented case due to the diffuse metastatic disease. Considering that there exists no standard treatment for NUT midline carcinoma until now [5, 27], the case was discussed interdisciplinary. A palliative systemical chemotherapy was started with nab-paclitaxel and carboplatin. This therapy is the first-line treatment of non-small cell lung cancer (NSCLC) in patients who are not candidates for curative surgery and/or radiation therapy [28].

In the presented case, the diagnosis and treatment of the tumor was complicated and delayed by the pregnancy of the patient. At the time the imaging of the thorax by MRI indicated a malignant disease with synchronic metastasis, the patient was in 31 4/7 weeks of gestation. Definite histological confirmation was achieved by bronchoscopic biopsy and the case then discussed interdisciplinary. The delay in further diagnostics with radiation exposure and cancer treatment of 3 weeks allowed the maturation of the fetal lung and was considered more beneficial in the palliative setting than an immediate cesarean section.

The pathological features of the tumor are also important for therapy. The NUT midline carcinoma in this case did not show the typical (15;19) translocation in fluorescence in situ hybridization (FISH) [16]. A monoclonal antibody to NUT genes was necessary to diagnose [18]. In these cases of a cryptic translocation, it is easy to miss a NUT midline carcinoma. This has already been reported in the case of a 13-year-old girl [29] as well as for a 28-year-old male with a misdiagnosed germ cell tumor [30]. Detailed pathological examination is important because a NUT midline carcinoma is mostly not suspected.

This report presents the case of a rare NUT midline carcinoma as tumor entity and emphasizes the need of interdisciplinary case discussions. Especially the additional factor of a pregnancy under this rare malignancy without specific therapy regimen until now had to be considered and required a highly personalized diagnostics and treatment planning. The case also illustrates that persistent or atypical symptoms should always be taken seriously and lead to supplementary diagnostics to exclude severe diagnoses.

## Data Availability

Data will be available upon request to the first author Sebastian Joel.

## Abbreviations

BET: Bromodomain and extra-terminal
MRI: Magnetic resonance imaging
PET-CT: Positron emission tomography-computed tomography
